# Quaternary Ammonium-Tethered Phenylboronic Acids Appended Supramolecular Nanomicelles as a Promising Bacteria Targeting Carrier for Nitric Oxide Delivery

**DOI:** 10.3390/polym14204451

**Published:** 2022-10-21

**Authors:** Yu Fang, Haiyan Cui, Xiaoqin Liang, Jianping Yu, Jianrong Wang, Guanghui Zhao

**Affiliations:** 1 State Key Laboratory of Applied Organic Chemistry, Institute of Biochemical Engineering & Environmental Technology, College of Chemistry and Chemical Engineering, Lanzhou University, Lanzhou 730000, China; 2 Department of Pathology, Gansu Provincial Hospital, Lanzhou 730000, China; 3 Gansu Provincial Maternity and Child-Care Hospital, Lanzhou 730050, China

**Keywords:** nitric oxide, targeted antimicrobial, wound healing

## Abstract

The delivery of drugs to focal sites is a central goal and a key challenge in the development of nanomedicine carriers. This strategy can improve the selectivity and bioavailability of the drug while reducing its toxicity. To ensure the specific release of nitric oxide at the site of a bacterial infection without damaging the surrounding normal tissue, we designed a host-guest molecule containing a host molecule with a target moiety and a nitric oxide donor to release nitric oxide. The boronic acid group in the structure of this nanoparticle interacts strongly and specifically with the surface of *E. coli*. In addition, the quaternary amine salt can interact electrostatically with bacteria, indicating a large number of negatively charged cell membranes; altering the molecular structure of the cell membrane; increasing the permeability of the cell membrane; and causing cytoplasmic diffusion and cell lysis, resulting in lethal activity against most bacteria. The synthesised molecules were characterised by ^1^H NMR and mass spectrometry. The strong specific interaction of the boronic acid moiety with the surface of *E. coli* and the electrostatic interaction of the quaternary amine salt with the cell membrane were confirmed by antibacterial experiments on molecules with and without the targeting moiety. The targeting group-modified micelles enhanced the antibacterial effect of the micelles very effectively through specific interactions and electrostatic interactions. In addition, in vitro skin wound healing experiments also confirmed the targeting and antimicrobial effect of micelles. These results suggest that the specific release of nitric oxide at the site of bacterial infection is an important guide to further address the emergence of antibiotic-resistant strains of bacteria.

## 1. Introduction

Microbial contamination caused by pathogens poses a serious threat to human health and has posed an unprecedented challenge to antimicrobial research in recent decades [1]. Usually, the use of antibiotics is the contemporary treatment of choice for bacterial infections. However, the misuse of antibiotics is inducing the emergence of more and more drug-resistant bacteria [2]. The antibiotic resistance crisis is one of the greatest challenges to global health, and if not actively addressed, it will have permanent and enormous humanitarian and economic consequences [3,4]. Notably, antibiotic resistance is associated not only with structural and genetic mutations in mayfly bacteria, but also with bacterial biofilms [5]. Therefore, the development of more effective antimicrobial agents, as well as the proposal of new antimicrobial strategies, are urgent needs for academic research and clinical treatment.

To date, many approaches with significant antimicrobial effects have emerged in various medical fields. Among these, nanomaterials technology offers powerful broad-spectrum antibacterial strategies. In contrast with the specific structures of antibiotics, NPs have diverse and variable characteristics, such as their chemical composition, surface functionalisation, shape, size, hydrophobicity or hydrophilicity, and many other intrinsically controllable variables. These characteristics facilitate the development of a variety of nanoantibacterial drugs with distinctive mechanisms of action from antibiotics [6]. The high surface area-to-volume ratio of NPs gives the drug new physical (e.g., mechanical, optical, and electrical) and chemical properties and high reactivity. It also allows close interaction between microbial membranes and the surface of NPs and easy penetration into the biofilm matrix and cell membranes, which in turn allows the surface functionalisation to flexibly modulate the antibacterial performance of NPs to achieve the desired antibacterial purpose [7]. NPs’ large specific surface area also makes them ideal drug-delivery vehicles, and compounds can often be immobilised on the surface of NPs by surface functionalisation to increase their solubility and targeted delivery [8,9]. Antibacterial nanomaterials have great potential to tackle bacterial infections because NPs can exert antimicrobial activity through a variety of mechanisms, such as direct interaction with the cell wall, the inhibition of biofilm production, the triggering of innate and adaptive immune responses, the generation of reactive oxygen species (ROS), the release of antimicrobial active substances, and the induction of intracellular reactions (interaction with DNA to inactivate intracellular active substances) [10,11]. 

Because free radicals play an important role in the fight against pathogenic microorganisms, recent research has focused on improving both the utilisation of free radicals in disease treatment and their biosafety. No studies have yet identified free radical-resistant strains, and therefore, there is a growing interest in free radical antimicrobial therapies, and researchers are looking for new free radical delivery platforms to achieve effective antimicrobial resistance. The inherent antimicrobial activity of free radicals in biochemical reactions can assist immune cells against most Gram-positive and Gram-negative pathogenic microorganisms and multidrug-resistant bacteria that have been isolated. They can also penetrate microbial membranes and thus exert antimicrobial activity against invading pathogens [12]. Although free radicals show great potential in antimicrobial applications, prevalent problems, such as short half-lives and poor controllability, remain a hindrance in antimicrobial therapy. Therefore, contemporary researchers have focused their efforts mainly on how to design free radical drug release precursors that can specifically release toxic free radical concentrations at the site of a bacterial infection in combination with the inflammatory properties of the bacterial infection without damaging the surrounding normal tissues [13]. Under such ideal conditions, a controlled free radical release and the effective delivery of free radical concentrations are of great guidance to further address the emergence of antibiotic-resistant bacterial strains and eliminate biofilm formation.

Current research related to the use of free radicals for antibacterial purposes can be categorised into two types of free radical antibacterial activity: reactive oxygen species (ROS) and reactive nitrogen species (RNS). Typical RNS include nitric oxide (NO), nitrogen dioxide (NO_2_), nitrosohydrogen (HNO), nitrite ion (NO_2_^−^), and peroxynitrite anion (ONOO^−^) [14]. RNS has stronger biocidal activity compared to ROS because it can exacerbate overall biological damage by triggering free radical peroxidation [15]. NO also plays an important role in physiological regulation (e.g., cardiovascular and nervous system messenger regulation). NO is an integral and highly conserved part of the host immune response, so few bacteria are able to escape the antibacterial effects of NO. In contrast, NO has two antimicrobial effects depending on its concentration. At low concentrations, NO acts as a signalling molecule, promoting the growth and activity of immune cells, whereas at high concentrations, NO covalently binds DNA, proteins, and lipids, thereby inhibiting or killing the target pathogen [12]. These factors have led to the widespread use of NO as one of the most valued members of the RNS family in antimicrobial and wound healing promoting treatments [14]. In biological applications, NO release donors are divided into low molecular weight NO donors and high molecular weight NO donors. Low molecular weight NO donors, such as organic nitrates, nitrites, metal–NO complexes, and S-nitrosothiols, have been shown to have significant antibacterial and antitumour activities and have great potential in the biological field. However, due to the presence of the non-specific targeted delivery of NO, the rapid systemic clearance of low molecular weight donors and concerns about non-essential toxicity have hindered the further clinical application of low molecular weight NO donors. In order to precisely deliver effective therapeutic concentrations to the lesion and reduce adverse effects, there is a need to develop NO drug release platforms that can be externally regulated and targeted.

Recently, there has been increasing interest in developing targeted antimicrobial materials and methods [16], which can effectively improve antimicrobial efficiency by applying targeted antimicrobial materials and strategies. For example, Nesha et al. designed a versatile and stimulating (NIR laser-activated) antimicrobial platform by combining the inherent photothermal capability and the excellent biocompatibility of polydopamine nanoparticles with the membrane targeting and cleavage activity of antimicrobial peptides (AMP) [17]. Cheng et al. proposed a new approach for targeting bacteria by using formic acid (a cellular metabolite found in certain bacterial species) to activate the antimicrobial drug and selectively inhibit its growth [18]. The synthesis of biocompatible enzyme-responsive silver nanoparticle assemblies and their application in the efficient targeting of methicillin-resistant Staphylococcus aureus for antimicrobial therapy has been demonstrated in a report by Yang et al. [19]. Herein, we designed functional nanomicelles particles with the dual-targeting delivery of nitric oxide antimicrobial agents to bacterial cell membranes, which can precisely target the cell membranes of Gram-negative bacteria. Quaternary ammonium salt (QAS) compounds have been shown to be powerful antimicrobial agents against spectroscopic bacteria. Bacterial surfaces are negatively and positively charged quaternary ammonium salts (QAS) can bind to negatively charged bacterial or biofilm surfaces through electrostatic interactions, altering the cell membrane molecular structure, increasing cell membrane permeability, which can even lead to cytoplasmic diffusion and cell lysis, and thus can be lethal to most bacteria [20,21]. Compared to commonly used antibiotic-based antimicrobial agents, QAS compounds are rarely resistant to bacteria [22]. However, Gram-negative bacteria, which consist of complex multilayered outer membranes, are difficult to be inserted and penetrated by QAS compound-based antimicrobial agents compared to Gram-positive bacteria that contain simple cell wall structures [23]. Consequently, achieving antimicrobial activity comparable to Gram-positive bacteria requires greater concentrations of QAS compound-based antimicrobial agents, increasing the toxic side effects arising from in vivo use [24]. Therefore, this challenge highlights the need for alternative platforms for effective control, effective targeting, or combination therapy. Boronic acid (BA), a compound with a boron centre connected to three hydroxyl groups through an oxy–boron bond, has been widely used in the development of biochemical sensors [25,26] and targeted antitumours [27,28] because of its high sensitivity for covalent binding to cis-diols in sugars. The rich lipopolysaccharide (LPS) on the cell wall surface of Gram-negative bacteria contains a cis-diol molecular structure, while the boronic acid group covalently couples to the cis-diol structure, and the boronic acid-based functional materials have been shown to have excellent recognition and targeting of bacterial properties [29,30]. Although phenylboronic acids can specifically recognize and target bacterial cell membranes, the antibacterial activity of nanoparticles modified by the phenylboronic acid groups with only a single antimicrobial mechanism is still insufficient for in vivo antimicrobial repair applications [31,32,33]. Therefore, the construction of a multimodal targeting therapeutic platform is a promising strategy to combat bacterial-associated infections.

**Scheme 1:**
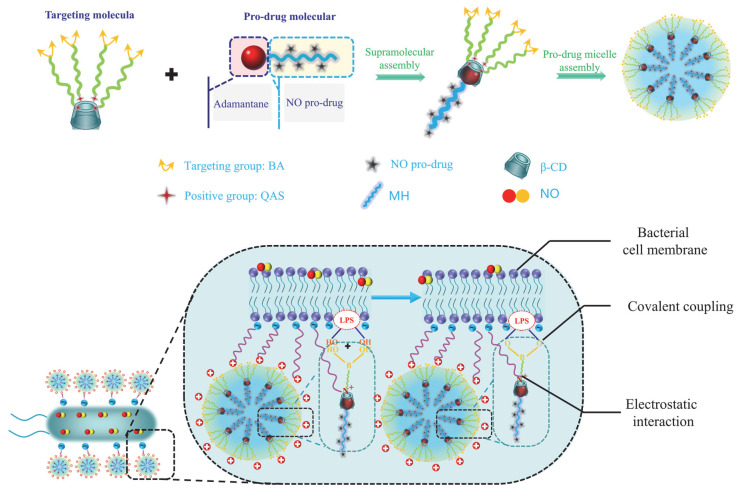
Schematic diagram of supramolecular assembly and targeted antibacterial mechanism.

Based on the construction of a multimodal targeted therapeutic platform to achieve a selective fight against bacterial-associated infections, we reported a targeted pro-drug nanoparticle with efficient antibacterial activity. In this study, a natural compound β-CD and its derivatives (maltoheptose) with good biocompatibility and abundant functional groups were selected as backbone molecules. Based on the host–guest interaction between cyclodextrin and adamantane molecules, an antibacterial nanomaterial that not only targets bacterial cell membranes, but also can release NO, was designed and synthesized. The antimicrobial process of the designed pro-drug nanoparticles is as follows(Scheme 1): pro-drug nanoparticles bind to the negatively charged outer membrane of bacteria with lipopolysaccharide (LPS) + NO production from the NO donor → bacterial apoptosis.

## 2. Materials and Methods

### 2.1. Materials

β-cyclodextrin (β-CD), triphenylphosphine (PPh_3_), bromomethylbenzeneboronic acid, p-methylbenzenesulphonyl chloride, silver nitrate (AgNO_3_), bromoacetyl bromide, sulfonamide, naphthylenediamine hydrochloride, 1-adamantaneamine hydrochloride, disodium terephthalate, sodium methanol, and pyridine were obtained from Shanghai Aladdin Reagents Ltd. (Shanghai, China). Ethyl ether anhydrous, acetone, methylene chloride, xylene, triethylamine, acetic anhydride, toluene, monomethyl iodine, 40% dimethylamine aqueous solution, sodium hydroxide (NaOH), dimethyl sulfoxide (DMSO), N,N-dimethylformamide (DMF), acetonitrile, 85% phosphoric acid aqueous solution, 98% hydrochloric acid, anhydrous ethanol, and anhydrous methanol were obtained from Leelanon-Bowa (Leelanon-Bowa Medicinal Chemicals Ltd., Tianjin, China). Dimethyl sulfoxide (DMSO) and N,N-dimethylformamide (DMF) were obtained by reduced pressure distillation CaH_2_ pretreatment. Deionized water was used throughout this study. Additionally, this study used Tegaderm™ Transparent Wound Dressing, which is a medical product sold and distributed by Minnesota Mining Manufacturing (Shanghai, China) International Trading Co. The Tegaderm sponge was purchased from China Jiangxi Xiang en Medical Technology Development Co. (Nanchang, China).

### 2.2. Measurements

The ^1^H NMR spectra were recorded on a JNM-ECS 400M NMR (JEOL, Akishima-shi, Japan) instrument using dimethyl sulfoxide-d6 (DMSO-d_6_) or deuterium oxide (D_2_O) as the solvent and TMS as the internal reference. The transmission electrons (TEM) were measured on a Talos F200C (FEI, Hillsboro, OR, USA). A mass spectrometric analysis was also performed on a Bruker Daltonics autoflex III smartbeam. Scanning electron microscopy was recorded on a Hitachi S-4800.

All animal experiments were conducted in compliance with the Animal Ethical Procedures and Guidelines of the People’s Republic of China and were approved by the Animal Ethics Committee of Lanzhou University (China) (No. 2022-G04).

## 3. Results and Discussion

In order to effectively improve the targeting performance, as well as synergistic and precise bactericidal anti-infective therapy, we report a nanoparticle with efficient antimicrobial targeting precursors. In this study, a natural compound β-CD and its derivatives (maltoheptose) with good biocompatibility and abundant functional groups were selected as backbone molecules. Based on the host–guest interaction between cyclodextrin and adamantane molecules, an antibacterial nanomaterial that not only targets bacterial cell membranes but also can release NO was designed and synthesized. NO can penetrate the membranes of bacteria and induce oxidative stress damage in bacteria without resistance to bacteria, and it is a widely used gaseous antibacterial agent. However, the high activity and the short half-life of NO have forced the development of various delivery systems to increase the local NO concentration and to achieve effective antibacterial activity. In the study, a quaternary ammonium-tethered phenylboronic acids appended supramolecular nanomicelle was constructed as a promising bacteria-targeting carrier for NO delivery. First, two targeting host molecules CD-QAS-B_7_ and CD-QAS-B_1_ with different numbers of dual-targeting functional groups were synthesized. Next, the guest molecules containing multiple NO donors (Ad-MH-NO) were prepared via nitrosation modification of maltoheptose. Finally, the amphiphilic coupled supramolecules containing hydrophilic and hydrophobic chain segments were assembled into supramolecular nanomicelles in water via host–guest interaction coupling.

### 3.1. Design, Synthesis, and Characterisation of CD-QAS-BA and Ad-MH-NO

**Figure 1:**
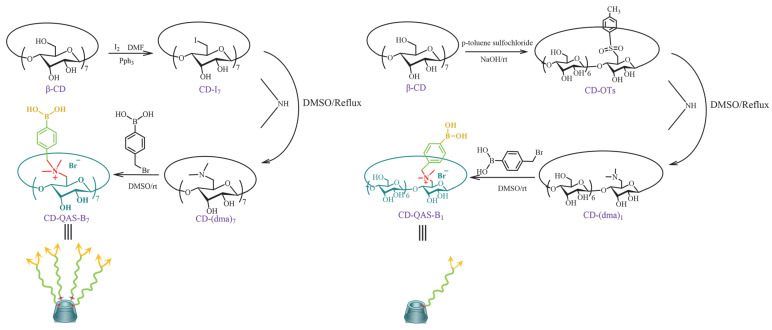
Synthetic route for CD-QAS-B.

**Figure 2:**
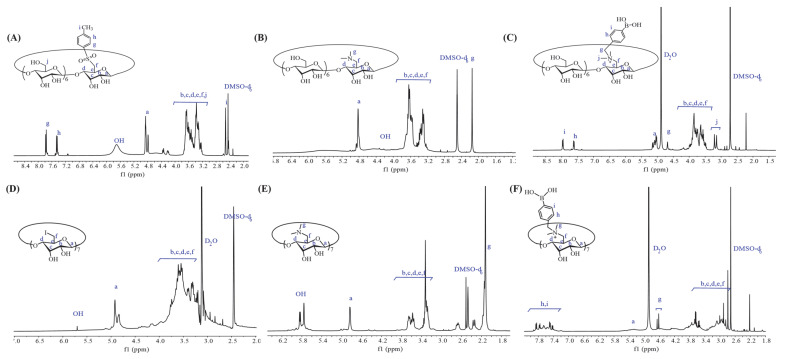
^1^H NMR spectra for (**A**) CD-OTs, (**B**) CD-(dma)_1_, (**C**) CD-QAS-B_1_, (**D**) CD-I_7_, (**E**) CD-(dma)_7_, (**F**) CD-QAS-B_7_.

**Figure 3:**
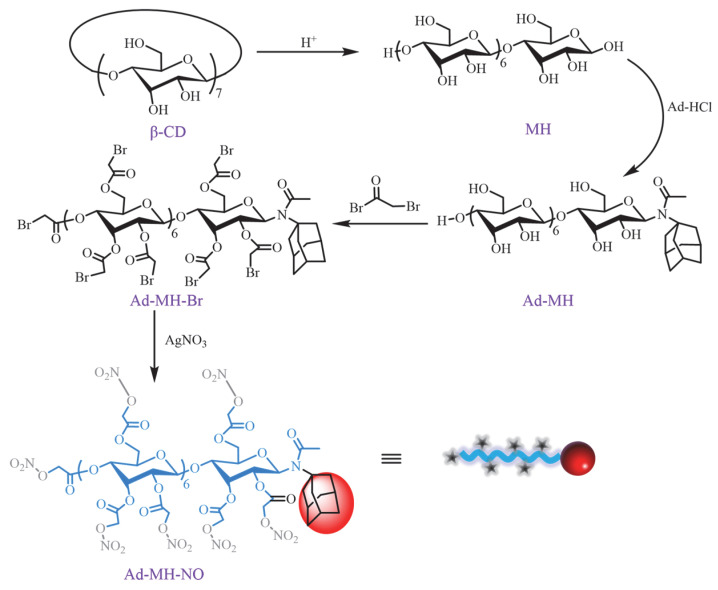
Synthetic route for Ad-MH-NO.

**Figure 4:**
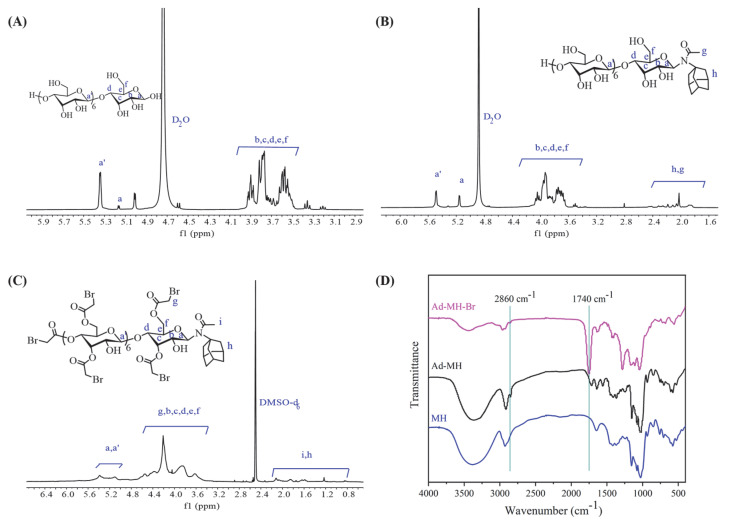
^1^H NMR spectra for (**A**) MH, (**B**) Ad−MH, (**C**) Ad−MH−Br, (**D**) IR spectra for Ad−MH/Ad−MH−Br/Ad−MH−NO.

The host molecules CD-QAS-B_7_ and CD-QAS-B_1_ with targeting groups were synthesized by a multistep synthesis, as shown in Figure 1. CD-QAS-B_1_ was synthesized by the substitution of the 6-hydroxyl group of β-CD with the p-toluenesulfonyl chloride and subsequent amination with the dimethylamine solution to give CD-(dma)_1_. CD-(dma)_1_ was reacted with 4-(bromomethyl) benzeneboronic acid to construct CD-QAS-B_1_ containing one target group. CD-QAS-B_7_ was synthesized by substituting the 6-hydroxyl group of β-CD with iodine, and subsequent amination with dimethylamine solution to obtain CD-(dma)_7_, which was reacted with 4-(bromomethyl) benzeneboronic acid to construct CD-QAS-B_7_ containing seven target groups. The molecular structures of the intermediates and the target molecules were verified by ^1^H NMR and MS spectra (Figure 2 and Figure S1). The experimental results showed that we successfully synthesized the dual-targeting host molecules CD-QAS-B_7_ and CD-QAS-B_1_. The preparation process of Ad-MH-NO is shown in Figure 3. First, maltoheptose (MH) was prepared by the ring-opening reaction of β-cyclodextrin, which provided the key sugar segment for the next step of the synthesis. Then, Ad-MH was obtained by modifying the adamantane amine to the reduced terminal hydroxyl group of MH, and the remaining modifiable hydroxyl groups were then reacted with acetyl bromide to obtain Ad-MH-Br with multiple alkyl bromides. Finally, the alkyl bromide was reacted with AgNO_3_ to obtain the Ad-MH-NO guest molecule containing multiple NO donors. The details of the synthesis of various molecules are in the Experiment Section (Supporting Information), and the molecular structures of the intermediates and the target molecules were verified by ^1^H NMR and FTIR (Figure 4).

### 3.2. Transmission Electron Microscopy (TEM) of Assembled Nanoparticles

**Figure 5:**
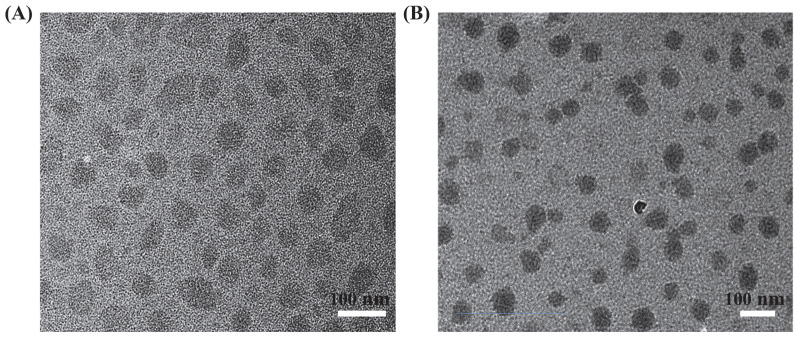
TEM images of Supramolecular nanomicelle (**A**) B_1_-QAS@MH-NO, (**B**) B_7_-QAS@MH-NO.

To obtain antibacterial materials with integrated targeting and NO donors, we assembled amphiphilic coupled supramolecules containing hydrophilic and hydrophobic chain segments into nanomicelles particles in water. As shown in Figure 5, the TEM images showed that the prepared nanomicelles had a uniform size and a well-dispersed circular structure, indicating that both of the main molecules of one and seven modified dual-targeting functional groups on cyclodextrins could couple with the guest molecules of NO donors to self-assemble and form regular nanostructures. Furthermore, CMC values were studied by the widely reported pyrene-probe based fluorescence technique. The ratio of the fluorescence intensity ratio of micelles I_403_/I_373_ to the logarithm of concentration is shown in Figure S2, the CMC value for CD-QAS-B1 was 1.175 × 10^−7^ mg/mL, the CMC value for CD-QAS-B_7_ was 5.754 × 10^−7^ mg/mL.

### 3.3. Detection of NO In Vitro

**Figure 6:**
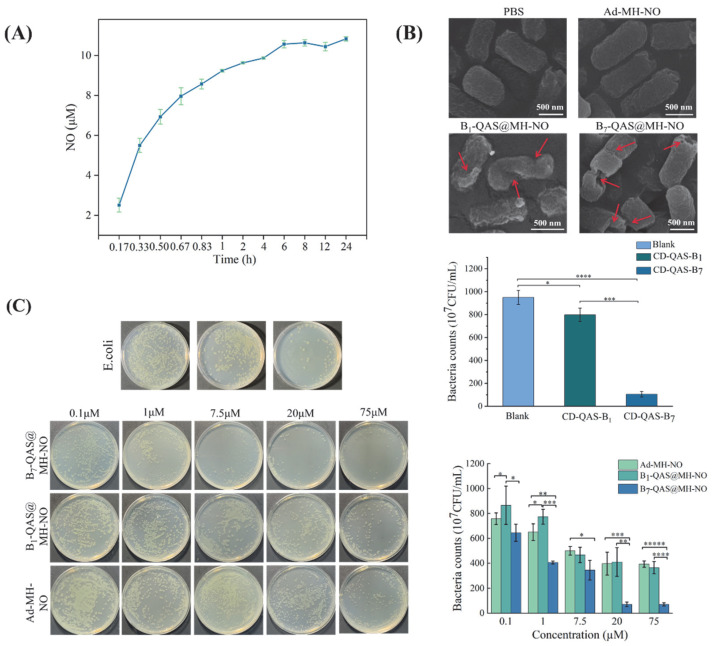
(**A**) The release of NO at 37 °C in PBS (pH 7.4) (**B**) SEM images of bacteria treated with different materials (red arrows indicate that the bacterial cell membrane was damaged) (**C**) Surviving colonies of bacteria on solid agar plates after interaction with PBS, CD-QAS-B_1_, CD-QAS-B_7_, B_7_-QAS@MH-NO, B_1_-QAS@MH-NO, Ad-MH-NO and its quantitative analysis. * *p* < 0.05, ** *p* < 0.01, *** *p* < 0.001, **** *p* < 0.0001, ***** *p* < 0.0001.

Real-time NO release was measured using a Griess reagent colour reaction. The kinetics of NO release from CD-QAS-B was determined under physiological conditions (pH 7.40, 37 °C). The initial concentration of NO can be obtained by quantifying the resulting colour by UV/VIS spectroscopy, and the resulting NO release is shown in Figure 6A. Overall, MH-NO has NO payload capability. NO release was most pronounced during the first few hours in PBS (pH 7.4) at 37 °C. The initial high concentration of bactericidal NO ensures an effective initial attack on microorganisms. As shown in Figure S3, the kinetic analysis of NO formation for all materials was found to be in accordance with the kinetic quasi first order equation.

### 3.4. Antibacterial Properties

Due to the specificity of the cell membrane structure of the Gram-negative bacteria, *E. coli* was chosen as the test subject of this work in order to investigate the antibacterial activity of our designed dual-targeting NO delivery system containing quaternary positive ions and boronic acid moieties. As shown in Figure 6C, the number of viable colonies of *E. coli* on solid LB agar plates varied after interacting with different materials for a period of time, respectively. Compared with the PBS solution control, although there was no functional group in Ad-MH-NO to target the bacterial cell membrane, the continuous release of NO also induced rapid apoptosis of bacteria, and thus the number of surviving bacterial colonies was much lower than that in the PBS control. When bacteria interacted with B_1_-QAS@MH-NO or B_7_-QAS@MH-NO dual-targeting nanoparticles, the results showed that the number of surviving bacterial colonies on the plates was much lower than that of the system without targeted property, and the number of colonies in the B_7_-QAS@MH-NO group was reduced by about half compared to the B_1_-QAS@MH-NO group. However, a large number of bacteria survived after interaction with cyclodextrins with a single target group. The results also showed that the dual-targeting functional groups could significantly enhance the antibacterial activity of the NO-delivered nanoplatform with the same NO content, and they were proportional to the number of targeting groups. As shown in Figure 6B, it can also be seen that the bacterial cell membrane integrity was barely visible after the interaction with Ad-MH-NO compared to the control group, while the bacterial cell membrane integrity was severely disrupted after the interaction with the NO delivery nanoparticles with targeting groups. This phenomenon is in line with our original idea of using the targeting strategy to increase the extent of NO damage to bacterial cell membranes in order to improve the antibacterial activity of NO.

### 3.5. Bacterial Staining Test

**Figure 7:**
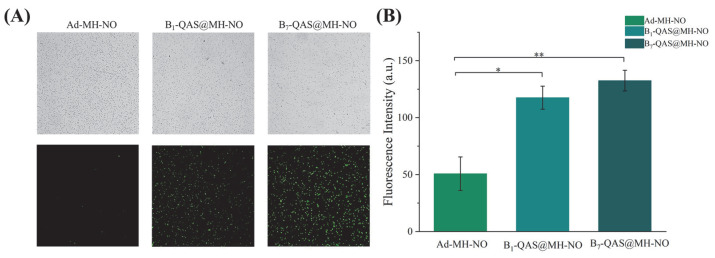
(**A**) NO fluorescence imaging in bright and dark fields, respectively, represent the bacteria after action with Ad-MH-NO, B_1_-QAS@MH-NO, B_7_-QAS@MH-NO. (**B**) The quantitative analysis of fluorescence intensity. * *p* < 0.05, ** *p* < 0.01.

DAF-FM can cross the membrane of living cells. After entering the cells, it can be catalysed by the cytoplasmic esterase to form DAF-FM, which cannot cross the cell membrane [34]. DAF-FM has very weak fluorescence, but after reacting with NO, it will produce the fluorescein benzotriazole, with an excitation wavelength of 495 nm and an emission wavelength of 515 nm. As shown in Figure 7, there was almost no fluorescence in the bacterial cell membrane after the interaction with Ad-MH-NO. Additionally, the fluorescence in the bacterial cell membrane after the interaction with B-QAS@MH-NO was significantly enhanced, and the fluorescence intensity was positively correlated with the number of the targeting groups. The stronger fluorescence in the bacterial cell membrane indicated that the additional NO-related content in the membrane could lead to serious damage to the bacterial cell membrane and could cause oxidative damage to DNA and liposomes, thus accelerating the bacterial death process. This result is consistent with the results of the in vitro antibacterial experiments described above, indicating that the dual-targeting NO delivery nanoplatform can effectively target bacterial cell membranes; deliver NO donors to the vicinity of bacteria for a cumulative release in a targeted manner; enhance local NO concentration; and thus improve the antibacterial activity of the nanoplatform.

### 3.6. Live/Dead Staining of Biofilms with 3D CLSM

**Figure 8:**
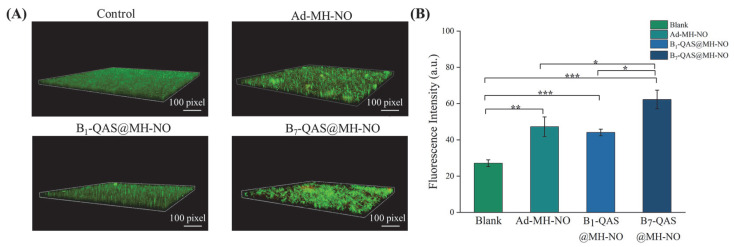
(**A**) Confocal laser scanning microscopy (CLSM) observation of *E. coli* films after incubation with PBS, Ad-MH-NO, B_1_-QAS@MH-NO, and B_7_-QAS@MH-NO. (**B**) The quantitative analysis of fluorescence intensity. * *p* < 0.05, ** *p* < 0.01, *** *p* < 0.001.

The live/dead staining of the biofilm was analysed using a three-dimensional confocal scanning microscope, as shown in Figure 8. While propidium iodide can reach the nucleus and embed DNA to produce red fluorescence that marks dead bacteria with damaged cell membranes, SYTO9 can enter all bacteria. In general, the fluorescence intensity of SYTO9 (green) decreases when both dyes penetrate the bacteria. Therefore, bacteria with intact membranes show green fluorescence, while bacteria with damaged membrane structure mainly show red fluorescence. Compared with PBS and Ad-MH-NO, a large number of red fluorescence spots appeared in B_1_-QAS@MH-NO and B_7_-QAS@MH-NO groups, indicating that the enhanced antibiofilm activity was based on the dual-targeting reaction.

### 3.7. Evaluation of Wound Healing Effect of Bacterial Infection

**Figure 9:**
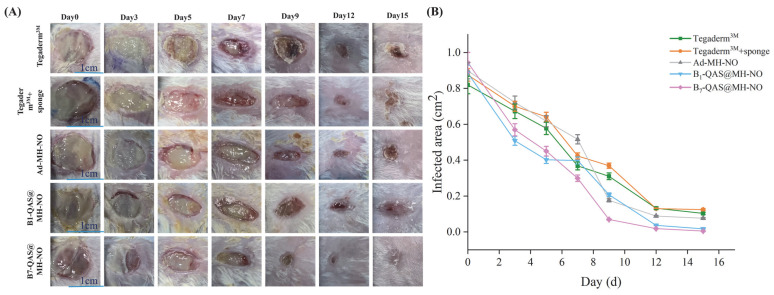
(**A**) Images of exposed abscesses treated with Tegaderm^3M^ (control), Tegaderm^3M^+ sponge, B_7_-QAS@MH-NO, B_1_-QAS@MH-NO, Ad-MH-NO and (**B**) Infected area at day 0, 3, 5, 7, 9, 12, and 15.

In order to further verify that the dual-targeting nanoparticles can promote wound healing in mice infected with *E. coli.* As shown in Figure 9, mice were divided into Tegaderm^3M^, Tegaderm^3M^+ sponge, B_7_-QAS@MH-NO, B_1_-QAS@MH-NO, and Ad-MH-NO groups, and wound healing was recorded at different times. Compared with the control group, the infected wounds of mice treated with B_7_-QAS@MH-NO and B_1_-QAS@MH-NO gradually formed scars and healed faster than the other three groups.

### 3.8. Histological Analysis

**Figure 10:**
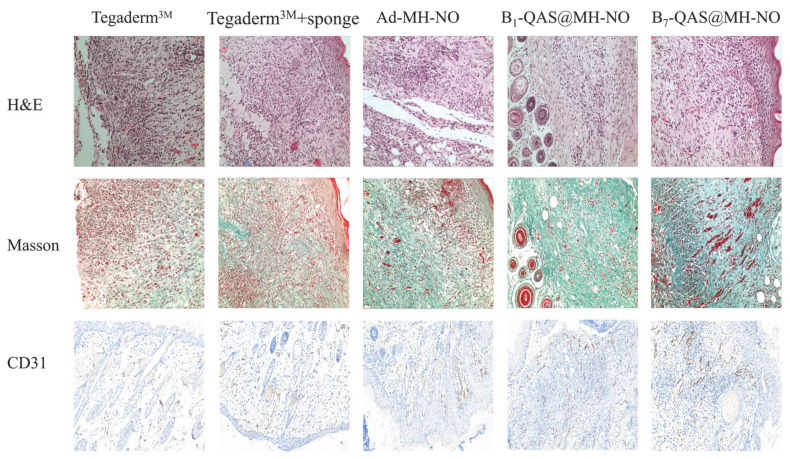
Histological micrograph of exposed abscess treated with Tegaderm^3M^ (control), Tegaderm^3M^+ sponge, B_7_-QAS@MH-NO, B_1_-QAS@MH-NO, Ad-MH-NO, Masson’s trichromatic staining, and immunohistochemical CD31 staining.

There are four overlapping steps in wound healing: inflammation, tissue formation, stroma formation, and remodelling. H&E, Masson, and CD31 were used for the histological analysis of infected wounds to study the subtle changes of the infected wounds. As shown in Figure 10, compared with the control group, inflammatory cells in the B_7_-QAS@MH-NO and B_1_-QAS@MH-NO groups were significantly reduced, indicating that the material could promote skin regeneration. Collagen synthesis by dermal fibroblasts is also important for wound healing. The results of the Masson trichromatic staining showed that the amount of collagen deposition on the wound surface in B_7_-QAS@MH-NO and B_1_-QAS@MH-NO groups gradually increased, and the intensity of collagen staining was significantly enhanced.

Immunohistochemical staining of endothelial cell marker CD31 showed that the expression of CD31 increased gradually and concentrated in the dermis, especially in the dermal papilla layer. The CD31 staining was more intense in the B_7_-QAS@MH-NO and B_1_-QAS@MH-NO groups, indicating that B-QAS@MH-NO stimulated angiogenesis more, promoted blood vessel formation, and aided nutrient transport in the process of wound healing.

## 4. Conclusions

In conclusion, we designed dual-targeting micelles loaded with NO and modified with phenylboronic acid and quaternary ammonium salts against cyclodextrins. We combined the specific interaction of the boronic acid moiety with *Escherichia* *coli* and the electrostatic interaction of quaternary ammonium salts with bacterial cell membranes, resulting in the specific release of nitric oxide at the site of bacterial infection. The results showed that micelles modified by the phenylboronic acid moiety with the quaternary ammonium salts effectively enhanced the binding and killing efficiency of these antimicrobial agents; exhibited adaptability and specificity in antimicrobial therapy; and had a low cytotoxicity. These results suggest that the specific release of nitric oxide from CD-QAS-B@MH-NO at the site of a bacterial infection may provide a new strategy for the construction of multimodal targeting therapeutic platforms.

## Data Availability

The data presented in this study are available on request from the corresponding author.

## Supplementary Materials

The following supporting information can be downloaded at: https://www.mdpi.com/article/10.3390/polym14204451/s1, Experiment Section and Figure S1: The MS spectra of (A) MH, (B) CD-OTs, (C) CD-(dma)_1_, (D) CD-(dma)_7_, (E) CD-QAS-B_1_, (F) Ad-MH; Figure S2: The CMC values for (A) CD-QAS-B1@MH-NO, (B) CD-QAS-B7@MH-NO; Figure S3: The Nitric oxide release kinetic curves at 37 °C in PBS (pH 7.4) for (A) Ad-MH-NO, (B) CD-QAS-B1@MH-NO, (C) CD-QAS-B7@MH-NO.
