# Supplementary material for: Quaternary Ammonium-Tethered Phenylboronic Acids Appended Supramolecular Nanomicelles as a Promising Bacteria Targeting Carrier for Nitric Oxide Delivery
Source: Polymers (Basel). 2022 Oct 21;14(20):4451. doi: 10.3390/polym14204451 (PMC9611690; doi:10.3390/polym14204451)
Supplement: Supplementary file 1 [file polymers-14-04451-s001.zip › polymers-1938860-supplementary.pdf]

polymers

Supporting Information

Quaternary Ammonium-Tethered Phenylboronic Acids Appended Supramolecular Nanomicelles as a Promising Bacteria Targeting Carrier for Nitric Oxide Delivery

Yu Fang [1,†], Haiyan Cui [1,†], Xiaoqin Liang [2], Jianping Yu [3], Jianrong Wang [3] and Guanghui Zhao [1,*]

[1] State Key Laboratory of Applied Organic Chemistry, Institute of Biochemical Engineering & Environmental Technology, College of Chemistry and Chemical Engineering, Lanzhou University, Lanzhou 730000, China

[2] Department of Pathology, Gansu Provincial Hospital, Lanzhou 730000, China

[3] Gansu Provincial Maternity and Child-Care Hospital, Lanzhou 730050, China

* Correspondence: zhaogh@lzu.edu.cn

† These authors contributed equally to this work.

Experiment Section

Synthesis of CD-OTs: Briefly, β-Cyclodextrin (6 g, 5.3 mmol) was dissolved in 120mL of sodium hydroxide solution(3.6 g, 0.09 mol)and then immersed in the 0-5°C ice bath.， until complete dissolution of CDs. P-toluenesulfonyl chloride (1.2 g, 6.3 mmol) was added dropwise into the mixture, followed by reacting 5 h at 0-5°C. The pH value of the crude product solution was adjusted to around 7.0, followed by putting in the 4°C fridge overnight. The precipitate was filtered and dried under vacuum for 2 d. The final product of CD-OTs was collected as white solid powder.

Synthesis of CD-(dma)1: CD-OTs (0.25 g，0.2 mmol) andKI(0.016g) was added into the single-neck round-bottom flask, followed by addition of 2mL of dimethylamine agueous solution(40%) until complete dissolution of the CD-OTs. The mixture was heated to 70°Cfor 7h,then cooled to room temperature and stirred overnight. The crude products were precipitated in 10 ml ethanol for 3 times and dried under vacuum at room temperature for 2d. The final product was white solid powder.

Synthesis of CD-QAS-B1: CD-(dma)1（0.0146 g，0.0125mmol）and Bromomethyl phenyl boric acid (1.5eq) was added into the single-neck round-bottom flask, followed by addition of 5mL of DMSO until complete dissolution of the CD-(dma)1. The mixture was carried out at room temperature under nitrogen atmospherehen for 24 h. The crude products were precipitated in cold acetone for 3 times and dried under vacuum at room temperature. The final product was white solid powder.

Synthesis of CD-(I)7: Triphenylphosphine (40 g，153 mmol) was dissolved in 160 mL of dimethylformamide (DMF). Iodine (40.5 g，160 mmol) and β-Cyclodextrin（11.5 g） was then added into the mixture. The solution was stirred at 70 °Cfor 18 h, then a large volume of DMF solvent was concentrated with a nitrogen stream. Afterwards, the solution changed from black brown to bright yellow suspension. by addition of 60 ml of 3 M sodium methanol solution . The mixture was stirred at room temperature for 30 min, followed by precipitation in Massive methanol solution. The precipitate was purified by

reflux in anhydrous methanol in a Soxhlet extractor. The precipitate dried under vacuum at room temperature for 3 d. The final product of CD-I7 was obtained as white solid powder.

Synthesis of CD-(dma)7: CD-(I)7 (0.6 g) was dissolved in 8 mL of dimethyl sulfoxide (DMSO) . 12mL of dimethylamine agueous solution(40%) was then added into the mixture. The solution was stirred at 70 °Cfor 48 h. After removing unreacted dimethylamine aqueous solution by rotary steaming ,followed by cold acetone for 3 times and dried under vacuum at room temperature.

Synthesis of CD-QAS-B7: CD-(dma)7（0.206 g）and Bromomethyl phenyl boric acid (2eq) was added into the single-neck round-bottom flask, followed by addition of 15mL of DMSO until complete dissolution of the CD-(dma)7. The mixture was carried out at room temperature under nitrogen atmospherehen for 24 h. The crude products were precipitated in cold acetone for 3 times and dried under vacuum at room temperature.

Synthesis of MH: β-CD(50g) and hydrochloric acid aqueous solution (200 mL 0.01M) was added into the 500 ml single-neck round-bottom flask, then the condensing reflux device is connected, and the decomposition continues to be stirred in the reflux state for 5 h . The pH value of the crude product solution was adjusted to around 7.0, followed by putting in the 4°C fridge overnight. Extraction of the filtrate to obtain a clear mixture, adding 0.35 ml of xylene solution to the mixture and stirring at 60 °C for 4 h, then putting in the 4°C fridge overnightt. Added an appropriate amount of diatomaceous earth to the above mixture for 0.5 h and then stand for 2 h, after the insoluble matter is removed by extraction, the mixture is concentrated to 1/10 of the original volume and then added 0.2 ml of xylene solution.The subsequent treatment steps are the same as above. Finally, after the mixture was concentrated to about 10 ml, it was added to a large amount of ethanol solution drop by drop, centrifuged and filtered to obtain a white malt heptasaccharide (MH) precipitate, and dried in vacuum at 40 °C.

Synthesis of AD-MH: 0.81 g 1-Adamantadine hydrochloride and 5 ml methanol were added to a 25 ml single-necked round-bottom flask for sonication, followed by 2 ml of triethylamine and 0.05 g MH in the reaction with magnetic stirring at room temperature for 3 days. After 2 washes with 30 ml of dichloromethane and dichloromethane:methanol (volume ratio 1:3), immediately add to the mixed solution containing 0.5 ml of acetic anhydride and 5 ml of methanol and continue stirring the reaction at room temperature for 2 days. The mixed solution changes from colorless to a faint rose red. After the all solvents were removed by steaming under reduced pressure, the toluene: methanol (volume ratio 1:1) mixed solution was added to the mixed solution and the unreacted acetic anhydride was removed by co-rotation, and the product was dried in vacuum.

Synthesis of Ad-MH-Br: 0.02 g Ad-MH, 0.03 ml of pyridine and 2.5 ml of dried DMF were added together in a 10 ml single-mouth round-bottom flask and dissolved by stirring. In addition, 0.04 ml of bromoacetyl bromide was dissolved into 0.5 ml of dried DMF, slowly added to the Ad-MH mixed solution under an ice bath, and added dropwise for 30 minutes. The reaction mixture reacted under a nitrogen atmosphere at room temperature for 18 h, and the solution changed from light yellow to dark brown and then to black and green. After the end of the reaction, it is precipitated with ether, and the ether is washed 2 times after vacuum drying at room temperature. The dried crude product was washed several times with secondary distilled water, and finally the product (Ad-MH-Br) was freeze-dried to give a white solid powder.

Synthesis of Ad-MH-NO: 0.35 g Ad-MH-Br, 0.44 g AgNO3 and 25 ml anhydrous acetonitrile were added sequentially to a 100 ml single-mouth round-bottom flask. React at 70 °C for 18 h under dark conditions and nitrogen atmosphere. AgBr was removed by spin centrifugation, the clear yellow-green solution was dialyzed in a mixed solution of

acetonitrile and water (volume ratio 1:1) for 24 h.Then dialyzed with distilled water for 24 h, and finally freeze-dried to give no supply precursor pale yellow-green powder solid.

Preparation of nanoparticles B7-QAS@MH-NO or B1-QAS@MH-NO: Dissolve 8 mg (CD-QAS-B7 or CD-QAS-B1) and 2 mg Ad-MH-NO in 1 ml DMSO. The mixed solution was sonicated for 5 h and then stirred magnetically at room temperature for 12 h. (wherein cyclodextrin and adamantane are in full contact effectively forming complexes). Then inject distilled water(10 ml) into DMSO mixed solution at a speed of 0.5 ml/h with a microsyringe pump.After injection, continue to stir for 12 h, the resulting product mixture solution is loaded into a dialysis bag (MWCO 2500 Da), dialyzed with distilled water for 24 h, and finally freeze-dried to obtain the final complexed self-assembled product. The entire complexing and assembly process takes place under light-protected conditions.

In vitro determination of NO: Ad-MH-NO, B1-QAS@MH-NO, B7-QAS@MH-NO were dissolved in buffer solution with pH 7.4, respectively. Measurement methods: The release behavior of NO was verified by Griess reagent at different time intervals.

In vitro antibacterial activity of B1-QAS@MH-NO and B7-QAS@MH-NO: the antibacterial activity was evaluated by plate counting method. In order to study the antibacterial effect of different concentrations of B1-QAS@MH-NO, B7-QAS@MH-NO, Ad-MH-NO on Escherichia coli, 106 CFU Escherichia coli suspension was treated with PBS and solutions of different concentrations. After incubation at 37°C for 12 h, the bacteria solution was diluted 10000 times with LB liquid medium. Spread the diluted solution (100 μl) on AGAR plate and incubate for 24 h to observe the bacteriostasis.

Bacterial morphology: The morphological changes of bacteria under different conditions were observed by scanning electron microscopy. In simple terms, bacterial cells are immobilized with 2.5% glutaraldehyde and then gradually dehydrated with a series of ethanol solutions. Finally, the dried bacterial cells were imaged by scanning electron microscopy.

Intracellular nitric oxide staining: Bacterial nitric oxide was stained with 3-amino-4-aminomethyl-2, 7-difluorescein diacetate (DAF-FM). After being treated in a constant temperature shaker at 37 °C for 2 h, the bacterial precipitate was collected by centrifugation, washed twice with PBS solution, and then dispersed into the DAF-FM DA diluted solution. The bacterial cell fluorescence imaging was observed under a fluorescence microscope at 37 °C for 30 min without light.

Live/dead bacteria staining analysis: Live/dead bacteria staining method was used to investigate the effects of different materials on the viability of bacteria. Specifically, the bacteria were mixed with a dye solution containing SYTO 9 and propidium iodide at room temperature for 0.5 h and then imaged with an inverted fluorescence microscope. According to the instructions, living cells are stained with SYTO 9 dye (green) and dead cells are marked with propidium iodide dye (red) for damage to cell walls and membranes.

Histological analysis: On the 15th day, the skin tissue of the infected wound was excised, fixed with 10% formaldehyde for 48h, and then cut into slices after paraffin embedding for histological analysis. Then, the anatomical skin tissues were stained with hematoxylin and eosin (H&E), Masson trichromatic staining, and IMMUNOHISTOchemical CD31 staining, respectively, to evaluate the actual effect of B-QAS@MH-NO system on bacterial eradication and healing of stagnant wounds in vivo.

Wound healing and in vivo inflammation experiments: All experiments were carried out in accordance with the Animal Ethics Regulations and Guidelines of the People's Republic of China and approved by the Animal Ethics Committee of Lanzhou University. In order to evaluate the antibacterial and anti-inflammatory effects of B-QAS@MH-NO on wound in vivo, a good wound model was established. Briefly, mice (6-8 weeks) were divided into five groups :Tegaderm3M(control group), Tegaderm3M + sponge, Ad-MH-NO, B1-QAS@MH-NO and B7-QAS@MH-NO, with five mice in each group. A skin wound with a diameter of 10 mm was established on the upper back and inoculated with E.coli (108 CFU, 20 µl) for 2 days.

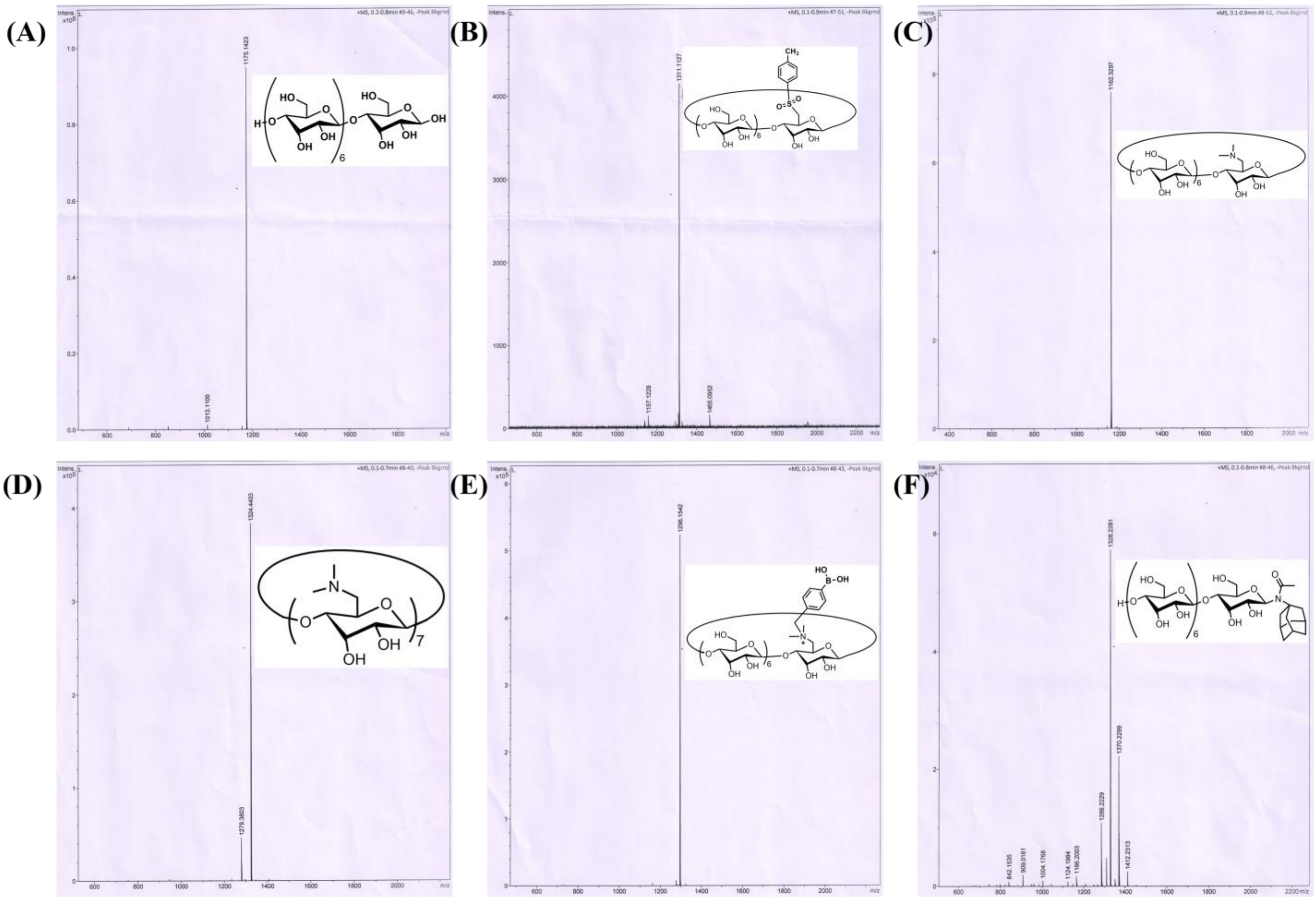

Figure S1. The MS spectra of (**A**) MH, (**B**) CD-OTs, (**C**) CD-(dma)$_1$, (**D**) CD-(dma)$_7$, (**E**) CD-QAS-B$_1$, (**F**) Ad-MH.

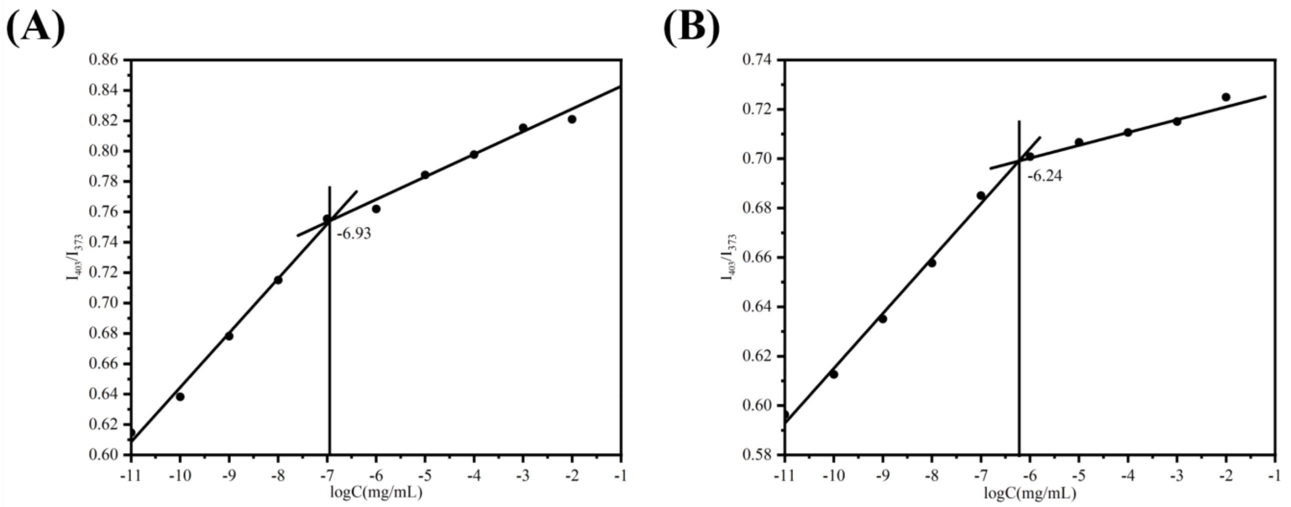

Figure S2. The CMC values for (**A**) CD-QAS-B1@MH-NO, (**B**) CD-QAS-B7@MH-NO.

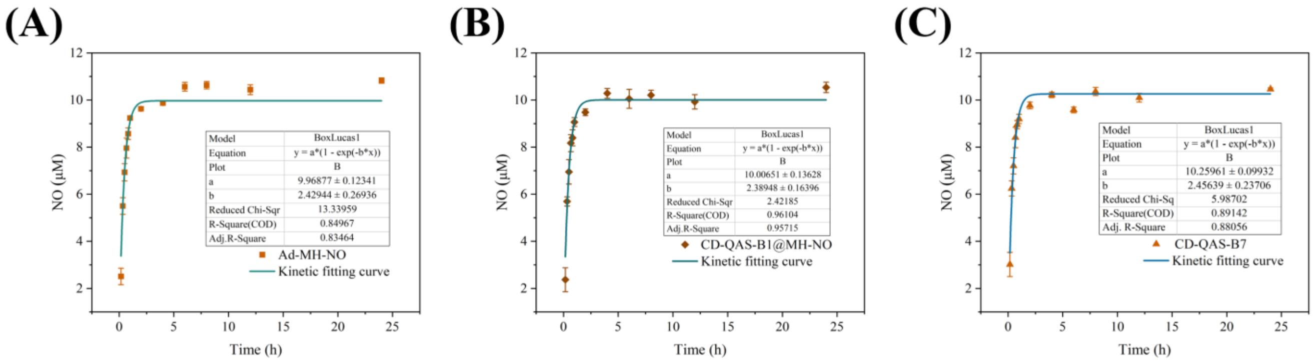

Figure S3. The Nitric oxide release kinetic curves at 37°C in PBS (pH 7.4) for (**A**) Ad-MH-NO, (**B**) CD-QAS-B1@MH-NO, (**C**) CD-QAS-B7@MH-NO.
